# MUC1 as a Putative Prognostic Marker for Prostate Cancer

**DOI:** 10.4137/bmi.s666

**Published:** 2008-05-14

**Authors:** Rona J Strawbridge, Monica Nistér, Kerstin Brismar, Henrik Grönberg, Chunde Li

**Affiliations:** 1 Departments of Oncology-Pathology; 2 Molecular Medicine and Surgery and; 3 Medical Epidemiology and Biostatistics, Karolinska Institutet, Stockholm, Sweden

**Keywords:** Mucin 1, prostate cancer, isoforms, antibodies, functional predictions

## Abstract

MUC1 is expressed on the apical surface of glandular epithelium. With functions including protection, adhesion and signaling, MUC1 has been implicated in prostate cancer. There are many splice variants, the best characterized of which are MUC1/1 and MUC1/2 which are determined by a SNP (rs4072037, 3506G>A).

Blood DNA from the general population, BPH, sporadic and hereditary prostate cancer subjects were genotyped for the rs4072037 SNP. G allele frequencies were significantly reduced in hereditary prostate cancer (15%) compared to population, BPH or sporadic prostate cancer samples (27%, 39% and 26% respectively). In addition, the G allele was lost from 3 of 8 heterozygous sporadic prostate tumor samples compared to matched blood DNA. Bioinformatics analysis of MUC1 protein sequences provides insight into differences between the variants which may be functionally relevant. The literature indicates discrepancies between immuno-histochemical studies, possibly due to the variety of MUC1 epitopes targeting diverse regions of the molecule. The contradictory findings in cell lines highlight the problem associated with inadequate experimental systems.

This is the first report of genetic differences in MUC1 between blood and prostatic cancer tissue. This finding is important as proof of principle, given that many association studies focus on blood DNA rather than on the tumor DNA. As yet, potential functional differences between splice variants has been paid little attention. Antibodies which discriminate between the variants and standardization of methods would help to clarify whether there is a role for MUC1 as a prognostic marker.

## Introduction

Mucin 1 (MUC1, also designated CD227, EMA, H23AG, MAM6, PEM, PEMT or PUM) is a large type I glycoprotein. Classically defined by the presence of an extensive variable number tandem repeat (VNTR), MUC1 moieties vary in size. Modifications, such as phosphorylation or glycosylation, are frequent on both the core protein and on the VNTR (Obermair et al. 2002). Indeed it is proposed that each repeat may have 5 glycosyl side chains added (Obermair et al. 2002). MUC1 is both a transmembrane and a secreted protein and it’s expression is restricted to the apical surface of glandular epithelium (Arai et al. 2005). MUC1 has roles in adhesion, protection from mechanical stress and bacterial infection, hydration and mucus production, immuno-supression and cellular signaling (Macao et al. 2006). Altered expression levels and localization, as well as delayed tumor formation being observed in knockout mice (Baruch et al. 1997), implicate MUC1 in cancers such as prostate cancer.

**Figure 1:**
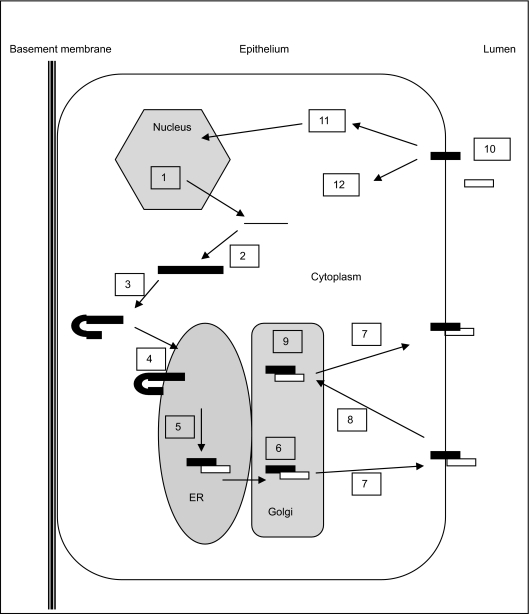
A schematic representation of MUC1 processing, adapted from (Julian et al. 2002; Engelmann et al. 2005; Levitin et al. 2005). 1) Transcription of MUC1 gene. 2) Translation of immature MUC1 protein. 3) Maturation of the initial MUC1 protein. 4) Trafficking of mature MUC1 into the ER. 5) Primary cleavage and dimerization. White fragments correspond to MUC1-N while black fragments correspond to MUC1-C. 6) Transport to golgi for post translational modifications i.e. glycosylation. 7) Trafficking to cell surface. 8) Recycling to golgi via clatherin-mediated endocytosis. 9) Post translational modifications i.e. sialylation. 10) Secondary cleavage releasing extra cellular component into intercellular space. 11) Signaling. 12) Endocytosis and recycling or degradation.

Processing of MUC1 proteins can result in both secreted and membrane-tethered variants, as demonstrated in Figure 1. The manner by which MUC1 undergoes cleavage has recently been described (Levitin et al. 2005; Palmai-Pallag et al. 2005; Macao et al. 2006), in which an enterokinase and agrin domain found in sea urchin sperm protein (SEA domain) generates the two polypeptides, MUC1-N and MUC1-C (Macao et al. 2006). The extra cellular fragment (MUC1-N) remains at the cell membrane by forming hetero-dimers with the transmembrane fragment (MUC1-C) (Palmai-Pallag et al. 2005). The functions of MUC1 are likely to depend upon the length and modifications of MUC1-N, as well as the localization and binding partners of MUC1-C.

**Figure 2:**
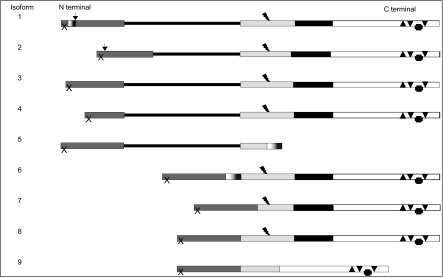
Schematic diagram of the 9 set a MUC1 variants. Solid black, transmembrane domain; pale grey, juxta-membrane extra cellular region; white, cytoplasmic region; dark grey, N terminal domain; solid line, VNTR; flash, SEA cleavage motif; arrow, SNP rs4072037; gradient grey, unique sequences; triangles (all), phosphorylation sites; cross, β catenin binding site; dotted area 9 amino acids unique to MUC1/2; X, signal peptide; arrow, exon 2 SNP.

There are as many as 9 different MUC1 variants (according to the SwissProt database, see Figure 2 for schematic presentation of variants), with varying degrees of post-translational modifications (PTMs). Functional differences or tissue-specific distribution of the variants have not been conclusively proven. The general format for identifying these proteins is MUC1/isoform name or number, for example MUC1/1 denotes MUC1 isoform 1. Variants may result from alternative splicing or genetic variations. The two best characterized variants, MUC1/1 or B (3506A) and MUC1/2 or A (3506G), are determined by a single nucleotide polymorphism (SNP) (rs4072037 (2005)) (Obermair et al. 2002) of MUC1 (chromosome 1q21). The variant allele (i.e. the least common) causes formation of a novel splice acceptor site (Ligtenberg et al. 1991) introducing an extra stretch of nucleotides between exons 1 and 2 of the mRNA, thus gives rise to MUC1/1 which encodes 9 amino acids not present in MUC1/2 (Ligtenberg et al. 1991). This SNP (G3506A, GeneBank Accession number NT_ 079484.1) is also associated, by linkage disequilibrium, with the length of the VNTR (Baruch et al. 1997). Differential expression of MUC1 variants, including MUC1/1 and MUC1/2, has been noted previously in ovarian (Obermair et al. 2002) and breast (Schmid et al. 2002) cancers.

The potential role of MUC1 in prostate cancer has been studied extensively. However, development of MUC1 as a biomarker for presence or progression of prostate cancer has been hindered by conflicting reports. This report provides experimental evidence of a reduced G allele frequency in hereditary prostate cancer as well as loss of heterozygocity (LOH) of MUC1 in prostate tumor DNA compared to matched blood DNA. In addition, *in silico* comparison of protein sequences and motifs and thus analysis of possible isoform differences is summarized.

## Materials and Methods

### DNA samples

Samples were collected for a previous study and genomic DNA extracted from blood as previously described (Li et al. 1999). 199 blood DNA samples were analyzed, including 46 from sporadic and 51 from hereditary prostate cancer patients, 35 from benign prostatic hyperplasia (BPH) patients and 67 from healthy young men (population sample). Of the 46 sporadic prostate cancer patients, 22 also had DNA samples extracted from prostate cancer tissues, forming 22 pairs of matched normal and tumor DNA samples. The baseline characteristics of all subjects are described in Table 1. The population samples were collected anonymously from healthy young men (age about 20 years) entering military service in the north of Sweden. Hereditary prostate cancer samples were collected from the same region, while BPH and sporadic prostate cancer samples were collected from, and thus are representative of, the greater Stockholm region. All samples were from Swedish subjects. The Swedish population is rather homogeneous, with approximately 85% being Caucasian. BPH patients were selected at random, but sporadic prostate cancer patients were selected due to large tumor size. All samples had matching slides, which were reviewed and diagnosis confirmed by a single pathologist at each centre. BPH samples represent a specific subset of the population which is very unlikely to subsequently develop prostate cancer, given the average age of 79 years. All BPH patients had histopathological examination of transurethral resection specimen to exclude possible incidental prostate cancer, in addition to measurement of serum prostate specific antigen (all within the normal range) and other clinical examinations (including ultrasound and digital rectal examination) without signs of prostate cancer. Hereditary prostate cancer is here defined as a patient having at least 2 first degree relatives with clinically and pathologically confirmed prostate cancer (Smith et al. 1996). Ethical permission from Karolinska Institutet and Umeå University has been granted.

### MUC1 SNP genotyping

Based on the human MUC1 genomic sequence (GeneBank accession number: NT_079484.1), we designed 2 primer pairs to carry out a nested PCR according to a standard PCR protocol using a Platinum Taq DNA polymerase (Invitrogen). Thermocycling parameters were set as follows: initial denaturing at 94 °C for 2 min; 30 cycles each of which at 94 °C for 30 sec, 53 °C for 45 sec, 74 °C for 45 sec; and a final extension at 74 °C for 5 min. The size of PCR product was confirmed by agarose gel electrophoresis, before being purified using a Qiaquick PCR purification kit (Qiagen) and quantified by photo spectrometry. A sequencing reaction was carried out using a sequencing primer on the reverse strand with a Beckman Coulter DTCS quick start kit in accordance with manufacturer’s instructions. Primer sequences are available upon request. Beckman Coulter’s CEQ™ 8000 Genetic Analysis System was used for sequence analysis. Quality control of sample processing was achieved by a single researcher performing all reactions, with a single protocol and kit. A random selection of samples was repeated to confirm results and accuracy. No clinical data was available to the researcher prior to genotype calling of these samples, thus preventing any bias. Sequence chromatograms were very clear (Fig. 2) and genotype calling was carried out by two independent researchers to confirm analysis.

### Statistical analysis

To determine whether specific alleles were associated with BPH or prostate cancer, comparisons were drawn on allele frequencies between sample sets using the Chi2 test, with the null hypothesis assuming that there is no significant difference between allele frequencies of each sample set.

### LOH analysis

Using the Beckman Coulter’s CEQ™ 8000 Genetic Analysis System, LOH analysis was carried out on sporadic prostate cancer samples by comparing the genotype of rs4072037 in blood DNA with tumor DNA from the same patient. Parameters for this analysis were set as follows: Percentage over peak spacing 70%; height ratio 30%; sensitivity 25%.

### In silico protein sequence analysis

MUC1 protein sequences (NCBI P15941, P15941-2, P15941-3, P15941-4, P15941-5, P15941-6, P15941-7, P15941-8 and P15941-9) were identified from the SwissProt database (Boeckmann et al. 2005), and (330608, 338983, 343482, 342814, 339690, 357374, 357375, 357377, 357378, 357580, 357381, 357383, all prefixed with ESNP00000) the Human Protein Atlas (Uhlen, 2007), and compared using cluster software T-coffee version 1 (Notredame et al. 2000) and Blast 2 (via NCBI website). Conserved motif sequences previously reported in literature (Palmai-Pallag et al. 2005; Kinlough et al. 2006) were manually identified in the protein sequences. Potential protein motifs were identified using SMART (Ponting et al. 1999) and MyHits motif scan platform (Falquet et al. 2002). The presence of signal peptides was assessed using the SignalP 3.0 platform (Bendtsen et al. 2004). PPSearch, via the EBI website, was used to detect potential protein motifs. The isoelectric point and molecular mass of the MUC1 protein variants were predicted using the Compute pI/Mw (Gasteiger et al. 2003) program.

## Results

### Experimental data

#### Disease association

MUC1 exon 2 (3506G/A) genotype frequencies in blood DNA samples only demonstrated significant differences (Table 2) when BPH and hereditary prostate cancer samples were compared. In contrast, allele frequencies were significantly different when hereditary prostate cancer (15% G allele) samples where compared to the population (27% G allele), BPH (40% G allele) or sporadic prostate cancer samples (27% G allele). Hereditary prostate cancer samples may be enriched for certain genetic variations, due to inclusion of multiple members of some families. When only one member of each hereditary prostate cancer family was included, significance still remained when compared to BPH samples, for both genotype and allele frequencies. That the significance was lost in some comparisons (hereditary prostate cancer vs. population and sporadic prostate cancer) may be due to the small number of G alleles present (n = 9).

#### Comparison between blood and tumor samples

**Figure 3:**
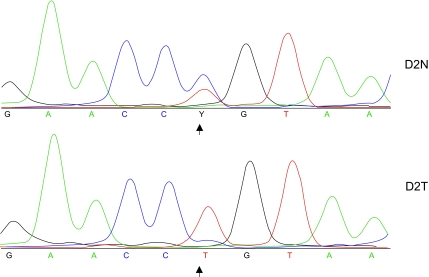
Chromatograms of blood (D2N) and tumor (D2T) samples from a prostate cancer patient. The MUC1 SNP site and its LOH in the tumor are indicated by an arrow at the corresponding base. Y indicates presence of both C and T alleles. Note that this is the reverse strand of DNA thus C and T correspond to G and A (respectively) on the forward strand.

8 of 22 pairs of normal and tumor DNA samples were G/A heterozygous in blood DNA samples. Of these 8 heterozygous patients, LOH could be clearly identified in 3 of the tumor DNA samples, with the G allele consistently being lost (Figure 3). Most DNA samples with matching tumour samples were of high Gleason grade, but this did not appear to differ much between genotypes (average for CC 8.5, TC 7, TT 7.9). There appeared to be no correlation between LOH and grade of tumour or age.

### Bioinformatics analysis of protein sequences

#### Variants

Table 3 provides a summary of differences between the MUC1 variants, including both described and bioinformatically predicted variants. There is a large degree of overlap between the different MUC1 variants and motifs, as shown schematically for the SwissProt variants (set a) in Figure 2. Another database, the Human Protein Atlas (HPA), lists 12 variants (set b). 4 of the SwissProt variants (6, 7, 8 and 9) share 99% homology with 4 of those listed by the HPA (19, 12, 3 and 17 respectively). Interestingly, with all 4 of these pairs, the one amino acid difference is the 4th from last amino acid, which in set a is alanine, but in set b is theonine. In addition, of the 12 variants in set b, only 2 contain a VNTR (2 and 16), and Blast2 analysis demonstrates 100% homology between these two variants.

**Figure 4:**
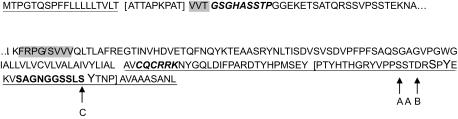
**Figure 4a** Conservation of MUC1 N terminal (MUC1-N) sequence. The sequence common to all variants is underlined. The signal peptide is the first 21 residues, inside of which the novel 9 aa [square brackets] are inserted as a result of the SNP (G or variant allele) in exon 2. The amino acids lacking from MUC1/3 and 4 (set a) are highlighted. A stretch of a further 10aa is present in all variants except MUC1/4 (shown in ***bold italics***). Continuation of the amino acid sequence is denoted by…. **Figure 4b** Partial protein sequence of MUC1 C terminal (MUC1-C), indicating predicted motifs. The binding site for β catenin is shown in **bold**, the SEA cleavage auto-catalytic domain is highlighted, with/indicating the residues between which cleavage occurs. Phosphorylation sites are indicated by larger font and arrows, with those targeted by GSK3β denoted A, Src denoted B and c-Abl denoted C. The sequence for palmitylation is shown in ***bold italics***. The sequence common to all membrane-tethered variants (thus excluding isoform 5) is underlined. The ERα interaction domain is enclosed in [square brackets]. Continuation of the amino acid sequence is denoted by….

Most variants share a similar primary structure, consisting of a signal peptide, an extra cellular domain, a transmembrane domain and a cytosolic domain. The N-terminal domains vary in length, however sequence alignment indicates that a stretch of 51 amino acids of the N terminal is common to all variants (Fig. 4a). MUC1/4 (set a) lacks a further 10 amino acids which are present in all other variants. Only variants 9 (set a), 11 and 17 (set b) lack a transmembrane domain. MUC1-C is also well conserved (Fig. 4b), with a stretch of 74 amino acids present in all but variants 5 (both sets). An adjacent stretch of 30 amino acids is common to all except variants 9 (set a), 11 and 17 (set b). The isoform sequences then become more divergent. Of note, both variants 5 (both sets) appear to be distinct from the other variants; they contain the N terminal consensus sequence, but at the C terminal share a stretch of only 23 amino acids, which contain no predicted domains. MUC1/5, 9 (set a) and 17 (set b) have two regions of 100% homology; the N terminal domain 54 residues, including the signal peptide and a stretch of 42 amino acids in the C terminal region.

#### Signal peptides

Protein sequences from both SwissProt (set a) and the HPA (set b) were analyzed. Signal peptides were predicted for the primary sequences of all variants in both sets (Bendtsen et al. 2004) and are present in the N terminal sequence (Fig. 4b). The signal peptide sequence reported previously (Baruch et al. 1997) is distorted in some variants, with 2 (set a), 11, 14 and 15 (set b) having a stretch of 9 amino acids inserted into the signal peptide (as a result of the SNP in exon 2 analyzed here) where as variants 3 and 4 (set a) are lacking 1 threonine and 2 valine residues. All other variants have the complete signal peptide.

#### Secreted vs membrane tethered variants

A SEA domain was predicted for most variants (Falquet et al. 2002), with the exception of 5, 7, 11, 15 and 19 (set b). However, searching the primary amino acid sequence of MUC1-C (Fig. 4b) of each isoform for the SEA autocatalytic cleavage motif (Palmai-Pallag et al. 2005) indicates that while most predictions are correct, MUC1/5, 9 (set a) and 17 (set b) do not have the consensus sequence. MUC1/19 (set b) has part of the consensus sequence, but as the first 2 residues of the motif are altered, it may not be functional (Palmai-Pallag et al. 2005).

#### Interactions

The motif conferring interaction with β catenin (Li et al. 1998) is present in the cytoplasmic domain (Fig. 4a), and is present in all but MUC1/5 (both sets). Variant 5 (both sets) are the only variants lacking the domain required for interaction with ERα (Wei et al. 2006).

#### Phosphorylation

Part of the cytoplasmic domain is common to all “membrane-tethered” variants (i.e. those containing a transmembrane domain), and there is further homology between some variants. The potential for cytoplasmic domain phosphorylation (as determined by manually checking the primary sequence for known motifs) by GSK3β, Abl and Src does not appear to vary between the variants, as demonstrated by Figure 4b, with only variants 5 (both sets) lacking these sites. Predicted phosphorylation by PKC and CK2 however demonstrates differences between the variants (Table 3). Of note, there are phosphorylation sites unique to variants 4 (set a) and 7 (set b).

#### Post-translational modifications

Interest in post translational modifications has mainly been focused on addition of glycosyl side chains, however many other modifications are likely. MUC1 variants 1–4 (set a), 2 and 6 (set b) are predicted to contain a proline-rich domain of 852 residues and 6 amino acids downstream, a serine-rich domain of 97 residues (Falquet et al. 2002). This region encompasses the VNTR. The lengths of the VNTRs of MUC1/3 and 4 (set a) have not been reported. Given the number of modifications possible in the VNTR the length of this region is likely to be very important for function. MUC1/5 (set a) also contains these two domains, but with the order inverted, and while the proline-rich domain is the same length, the serine-rich domain is only 47 amino acids long.

A potential motif for palmitylation (Kinlough et al. 2006) is found in the cytoplasmic region (Fig. 4b and Table 3), thus is predicted for all variants except MUC1/5 (both sets).

Glycosylation of MUC1 variants is dependent on the glycosyl transferases in the cell, however primary sequence analysis does indicate potential sites for attachment. Most side chains are added to the VNTR. Each repeat of the VNTR has the potential for 5 glycosyl modifications. Variants 6–9 (set a) and most of set b variants do not contain the repeats characteristic of mucins, thus are unlikely to be modified to the same extent. A variety of modification sites are predicted for all variants of MUC1 (Table 3). Potential myristylation sites indicate another modification, which all membrane-tethered variants share (Table 3). Of note, variants 15 and 7 (set b) have unique modifications sites, for glycosylation and myristylation respectively. Patterns of possible sialylation modifications may have similarities with those seen in glycosylation, in that the VNTR is the primary site for such additions.

## Discussion

The results presented here demonstrate, for the first time, a significantly reduced frequency in blood DNA of the MUC1 3506G allele in hereditary prostate cancer compared to population, BPH and sporadic prostate cancer samples. The same 3506G allele is subject to LOH in prostate cancer samples compared to matched blood samples. Differential expression of MUC1 variants 1 and 2 is thus implicated in prostate cancer. Loss of the G allele leads to a switch in expression from both MUC1/1 and MUC1/2 to exclusively MUC1/2, thus a protein with an intact signal sequence and shorter VNTR. A shorter VNTR may lead to a decreased protective function of this Mucin on the normal prostatic epithelial cell. Moreover, as motifs for PTMs are mainly present in the VNTR, the length of VNTR may theoretically have a multitude of effects on protein function. Due to the limited size of the sample set, independent validation is required. However, this finding is important as proof of principle, given that many association studies focus on blood DNA rather than on the tumor DNA.

Diagnostic and prognostic use of differentially expressed MUC1 variants has been reported for ovarian and breast cancers (Obermair et al. 2002; Schmid et al. 2002). As yet, this has not been addressed in prostate cancer. Our genotyping of matched tumor and blood samples indicates that isoform differences are likely equally important in prostate cancer. For example, the SNP *per se* may not be critical for function, as it does not alter the encoded amino acid. The functional difference is likely to stem from the associated VNTR lengths and potentially from the altered signal peptide sequence caused by the effect of the SNP on splicing. The significant reduction in frequency of the G allele of MUC1 in hereditary prostate cancer compared to the general population, BPH and sporadic prostate cancer, and the trend of reduction of the G allele in sporadic prostate cancer compared to the general population and BPH is intriguing and worth further investigation. The influence of different environmental exposures acting upon the hereditary prostate cancer and general populations compared to the sporadic prostate cancer and BPH subjects can not be ruled out.

MUC1 is a complex gene from which a number of variants can potentially be spliced with further complex PTMs. This makes it difficult to pinpoint the functional significance of the allelic variation of the SNP presented here (rs4072037). Therefore we have analysed *in silico* predictions of MUC1 variants. The potential for differences between the variants has not previously been assessed. Most of the variants are *in silico* translations of predicted mRNA splicing/open reading frames. Only variant 7 (set a) is reported to be specific to cancer (Obermair et al. 2002), while some are reportedly differentially regulated between normal and malignant tissue. The domains and sites for PTMs predicted are theoretical, thus biological evidence of their importance for the function of these variants is still required. However, this analysis provides insight into differences between the variants which may be functionally relevant.

A signaling role for MUC1 has been proposed. MUC1-C has been shown to colocalize with both ERα and P53 (Wei et al. 2005; Wei et al. 2006), and influences stability or transcriptional activity of its binding partner. Sub-cellular localization of the protein is likely to determine interactions of this manner.

The implications of the differences in MUC1 signal peptides are unknown. Inserting or losing amino acids may render the sequence inactive, or may enhance its function. It would be interesting to elucidate whether there are differences in processing efficiency or sub-cellular localization as a result of the variations in this region. Variants lacking the transmembrane domain will not, unlike the classical mucin, be inserted into the membrane, however whether they are secreted, degraded or remain in the cytosol where it might be more readily available for signaling, is not yet known. Variants which lack the TM domain yet contain a complete signal peptide (variants 9 (set a), 11 and 17 (set b)) may be secreted rather than incorporated into the cell membrane. This possibility is intriguing as the only isoform confirmed to be secreted (variant 5 (set a)) contains a VNTR. The absence of the VNTR from variants is likely to influence their steric barrier function the most, and may influence ligand-receptor-like interactions between MUC1-N and MUC1-C fragments and potentially signaling mechanisms. Due to the SEA domain, most variants are potentially able to give rise to a soluble extra-cellular domain. As MUC1/5 (set a) is a secreted isoform the lack of the SEA domain is unsurprising, and may suggest that isoform 9 (set a) may also be secreted. Variants which give rise to extra cellular fragments or are secreted may function as ligands for the membrane tethered potential receptors.

Differences in phosphorylation status of the cytoplasmic domain may determine signaling pathways utilized by the different variants. In particular the presence of PKC phosphorylation sites unique to variants 6 (set a), 7 and 19 (set b) are of interest, in light of an association between PKC phosphorylation and anchorage independent growth (Thompson et al. 2006). Likewise, a CK2 phosphorylation site unique to variants 4 (set a) and 7 (set b) could alter signaling. Thus, the variants may differ in their oncogenic potentials. Further research into the mechanisms and specificity of modifications and their association with progression is awaited with interest.

Most studies of MUC1 focus on the roles which occur by virtue of their VNTR domains, such as adhesion, mucus production and barrier function. Given the importance of the VNTR domain, it is interesting that the variants lacking this domain also contain the SEA site, implying that they too may give rise to soluble extra cellular fragments. These variants would give rise to very short extra cellular fragments as well as transmembrane fragments. How the two fragments transduce signals between the exterior and interior cellular environment is not clear. For example, ligand-receptor-like interactions between secreted and transmembrane MUC1 variants which result in phosphorylation of the MUC1/6 tail have been reported (Baruch et al. 1999; Obermair et al. 2002). This poses the questions: is it only the variants which lack a VNTR which are able to act as receptors, or do the trans-membrane fragments of all variants have this function? Similarly, do all extra cellular fragments or variants have potential as ligands? The differences in the variants ability to form either ligands or receptors and specificity of potential interactions add another level of complexity to functions of MUC1. It is feasible that the relative abundance at the cell surface of the different variants and competition between variants may determine the resulting signals.

That both under and over expression of MUC1 is associated with prostate cancer death (Andren et al. 2007) suggests that the level of VNTR-containing MUC1 variants is regulated in normal cells to maintain a precise level. However, the possibility that variations in expression of non-VNTR-containing variants may determine tumorigenic potential can not be excluded. Regulation of glycosylation and other modifications is a dynamic process (Julian et al. 2002) and may determine the adhesive properties of the molecule and thus it’s ability to interact with other cell types. In addition, a heavily glycosylated VNTR would provide a mesh which may store growth factors and minimize immune or pathogen interactions with the cell membrane. The study reporting an association between glycosylation and angiogenesis but not PSA level or Gleason grade (Papadopoulos et al. 2001) suggests that MUC1 can indirectly (in this case via neovascularization) influence tumor growth. Further, it was previously reported that sialyated MUC1 interrupts cell-cell adhesion, but removal of sialyation restored adhesion (Wesseling et al. 1996), thus the modifications are functionally relevant.

Table 4 summarizes the variants likely to have been detected by the different antibodies in the immunohistochemistry studies reported to date of MUC1 expression in prostate cancer. The conclusions of these studies are thus limited to the variants detected by each antibody. Results of these studies have been inconclusive or contradictory and only one addressed the problem of multiple variants, if only briefly. Furthermore, contradictory results in prostate cancer cell lines have also been observed when PC3, DU145 and LNCAP (or a sub culture, LNCaP LN3) were analyzed, with either differential or no expression observed (O’Connor et al. 2005), compared to high levels of over expression in all cell lines (Cozzi et al. 2005). The specific epitopes recognized by some antibodies used are unclear and may be part of the reason for the disparity in the results between studies. Antibodies which discriminate between the variants are required for reliable assay of protein expression. Another reason for contradictions may be the presence of functionally different variants, so that measurement of total MUC1 expression may not be conclusive.

Most studies have assessed expression of MUC1 by targeting antibodies to the VNTR, thus fail to detect expression of 4 of 9 MUC1 set a (and all of set b) variants. Indeed, the modifications added to the VNTR are highly variable and dynamic, thus a negative result when using an antibody which recognizes this region may be misleading, as variations in modification patterns (such as glycosylation) are likely to alter binding specificity. In addition, the VNTR can be extensive, so the question remains as to whether dense staining of a tissue sample reflects antibody molecules binding to multiple distinct MUC1 molecules, or many antibody molecules binding to the VNTR of a single MUC1 peptide.

We believe that the value of assessing variations in isoform expression has, so far, been under appreciated and methods to date lack standardization which would allow for meta-analyses of results. Predictions of differences between the MUC1 variants suggest distinct functions, of as yet unknown importance. The actual expression of *in silico* predicted variants needs to be confirmed and their biological functions determined experimentally. Further research into the mechanisms and specificity of PTMs and their association with progression is awaited with interest. Improvement in methods for determining isoform expression patterns and functions could thus yield valuable information. The finding described in this report of a prostate cancer-associated functionally relevant variation supports the notion that the MUC1 gene may be a useful marker for prostate and other cancers.

## Figures and Tables

**Table 1:** Clinical characteristics of sample sets.

		Population	BPH	SPC	HPC
n		67	35	46	51
Age (years)	Mean age	20	79	69	67
	sd		3.8	8.4	8.4
Stage	Localized			28	27
	Advanced			30	15
	Metastatic			6	0
	(n)			46	42
Grade	Grade 1			9	14
	Grade 2			14	0
	Grade 3			22	31
	(n)			45	45

BPH benign prostatic hyperplasia SPC sporadic prostate cancer; HPC hereditary prostate cancer; Localized Stage T1-2 Advanced T3-4, Metastatic TxN1Mx or TxNxM1; grading as per WHO, where 1 well differentiated (approximately Gleason grade 2–6), 2 medium differentiation (approximately Gleason grade 7), 3 poorly differentiated (approximately Gleason grade 8–10).

**Table 2:** MUC1 SNP (rs4027037) allele frequencies in BPH, sporadic and hereditary prostate cancer and population sample sets.

	Genotype			Total	Chi2 vs population	Chi2 vs BPH	Chi2 vs SPC	Allele				Total	Chi2 vs population	Chi2 vs BPH	Chi2 vs SPC
	GG	GA	AA					G	%	A	%				
Control	9	18	40	67				36	27	98	73	161			
BPH	6	15	13	34	0.116			27	40	41	60	108	0.063		
SPC	4	18	26	48	0.406	0.260		26	27	70	73	123	0.975	0.089	
HPC	3	10	40	53	0.157	**0.002**	0.076	16	15	90	85	121	**0.028**	<**0.001**	**0.036**
HPC*	2	5	22	29	0.308	**0.011**	0.142	9	16	49	84	74	0.088	**0.003**	0.097

BPH benign prostatic hyperplasia; SPC sporadic prostate cancer; HPC hereditary prostate cancer; HPC*, only one member of each family included; Significant values (p < 0.05) highlighted in **bold**.

**Table 3:**
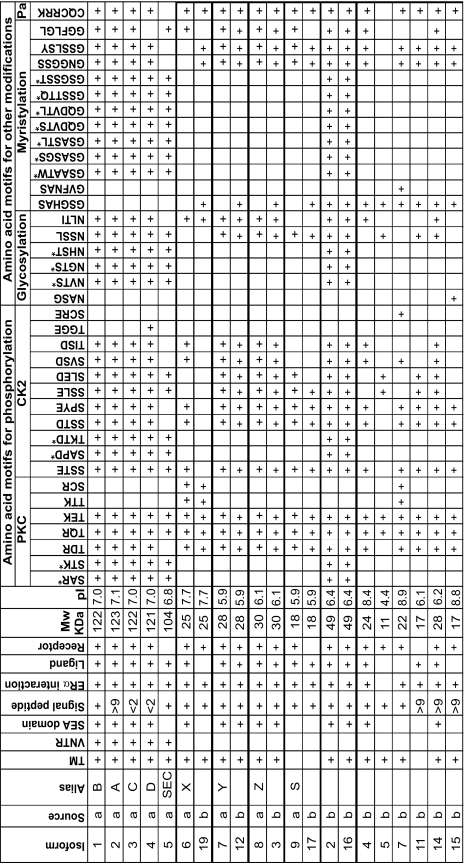
Predicted functions, characteristics and sites of post translational modifications of MUC1 variants.

Where splice variants are from a SwissProt, b Human Protein Atlas. TM transmembrane domain, VNTR variable number tandem repeat, Mw molecular weight, pI isoelectric point, PKC protein kinase C, CK2 casein kinase 2, Pa palmitylation, > extra amino acids (compared to MUC1/1), < loss of amino acids, ¤ potential to act as a receptor or ligand, * most likely in the VNTR.

**Table 4:** Summary of MUC1 antibodies used in studies of prostate cancer (PubMed search terms “MUC1, expression, prostate, cancer”).

Antibodies	Isoforms	Epitope	Clonality	References
139H2	N/A	N/A	mab	(Ho et al. 1993)
214D4	1,2,3,4,5	VNTR	mab	(O’Connor et al. 2005)
B27.29	1,2,3,4,5	VNTR/hyper	mab	(Kirschenbaum et al. 1999; Schut et al. 2003; Burke et al. 2006)
BC2	1,2,3,4,5*	core/hypo	mab	(Burke et al. 2006)
BrE3	1,2,3,4,5*	core/hypo	N/A	(Burke et al. 2006)
C595	1,2,3,4,5	VNTR	mab	(Mitchell et al. 2002; Cozzi et al. 2005)
CT-1	1,2,3,4,6,7,8	cytoplasmic	N/A	(O’Connor et al. 2005)
DF3	1,2,3,4,5,6,7,8,9	N terminal	mab	(Ho et al. 1993)
EMA	1,2,3,4,5*	core/hypo	N/A	(Burke et al. 2006)
H23	1,2,3,4,5	VNTR	N/A	(Legrier et al. 2004)
HMFG-1	1,2,3,4,5	VNTR/hyper	mab	(Mitchell et al. 2002; Schut et al. 2003; Burke et al. 2006)
HMFG2	1,2,3,4,5	VNTR	mab	(Mitchell et al. 2002; Zhang et al. 2004; Singh et al. 2006)
M8	N/A	N/A	N/A	(Legrier et al. 2004)
MoAb	1,2,3,4,5#	glycosylated	mab	(Papadopoulos et al. 2001)
MY.1E12	1,2,3,4,5#	sialyated	mab	(Arai et al. 2005)
NCL core	1,2,3,4,5	core/hyper	N/A	(Burke et al. 2006)
sc7313	1,2,3,4,5	VNTR	mab	(Lapointe et al. 2004; Andren et al. 2007)
SM3	1,2,3,4,5	VNTR/hypo	mab	(Mitchell et al. 2002; Burke et al. 2006)
VU4H5	1,2,3,4,5	VNTR	mab	(Andren et al. 2007)

* Assuming core refers to the non-VNTR region of the extracellular domain.

# Most side chains are added to the VNTR. mab monoclonal antibody, pab polyclonal antibody, hypo hypoglycosylated, hyper hyperglycosylated N/A not available.

## References

[b1-bmi-03-303] Andren O Fall K Andersson SO 2007 MUC-1 gene is associated with prostate cancer death: a 20-year follow-up of a population-based study in Sweden Br. J. Cancer 97 6 730 4 17726465 10.1038/sj.bjc.6603944 PMC2360377

[b2-bmi-03-303] Arai T Fujita K Fujime M 2005 Expression of sialylated MUC1 in prostate cancer: relationship to clinical stage and prognosis. Int. J. Urol 12 7 654 61 16045558 10.1111/j.1442-2042.2005.01112.x

[b3-bmi-03-303] Baruch A Hartmann M Yoeli M 1999 The breast cancer-associated MUC1 gene generates both a receptor and its cognate binding protein. Cancer Res 59 7 1552 61 10197628

[b4-bmi-03-303] Baruch A Hartmann M Zrihan-Licht S 1997 Preferential expression of novel MUC1 tumor antigen isoforms in human epithelial tumors and their tumor-potentiating function Int. J. Cancer 71 5 741 9 9180140 10.1002/(sici)1097-0215(19970529)71:5<741::aid-ijc9>3.0.co;2-r

[b5-bmi-03-303] Bendtsen JD Nielsen H von Heijne G 2004 Improved prediction of signal peptides: Signal P 3.0. J. Mol. Biol 340 4 783 95 15223320 10.1016/j.jmb.2004.05.028

[b6-bmi-03-303] Boeckmann B Blatter MC Famiglietti L 2005 Protein variety and functional diversity: Swiss-Prot annotation in its biological context. CR. Biol 328 10–11 882 99 10.1016/j.crvi.2005.06.001 16286078

[b7-bmi-03-303] Burke PA Gregg JP Bakhtiar B 2006 Characterization of MUC1 glycoprotein on prostate cancer for selection of targeting molecules. Int. J. Oncol 29 1 49 55 16773184

[b8-bmi-03-303] Cozzi PJ Wang J Delprado W 2005 MUC1, MUC2, MUC4, MUC5AC and MUC6 expression in the progression of prostate cancer Clin. Exp. Metastasis 22 7 565 73 16475027 10.1007/s10585-005-5376-z

[b9-bmi-03-303] Engelmann K Kinlough CL Muller S 2005 Transmembrane and secreted MUC1 probes show trafficking-dependent changes in O-glycan core profiles Glycobiology 15 11 1111 24 15972891 10.1093/glycob/cwi099

[b10-bmi-03-303] Falquet L Pagni M Bucher P 2002 The PROSITE database, its status in. Nucleic Acids Res 30 1 235 8 11752303 10.1093/nar/30.1.235 PMC99105

[b11-bmi-03-303] Gasteiger E Gattiker A Hoogland C 2003 ExPASy: The proteomics server for in-depth protein knowledge and analysis. Nucleic Acids Res 31 13 3784 8 12824418 10.1093/nar/gkg563 PMC168970

[b12-bmi-03-303] HapMap 2005 www.hapmap.org

[b13-bmi-03-303] Ho SB Niehans GA Lyftogt C 1993 Heterogeneity of mucin gene expression in normal and neoplastic tissues. Cancer Res 53 3 641 51 7678777

[b14-bmi-03-303] Julian J Carson DD 2002 Formation of MUC1 metabolic complex is conserved in tumor-derived and normal epithelial cells. Biochem. Biophys. Res. Commun 293 4 1183 90 12054500 10.1016/S0006-291X(02)00352-2

[b15-bmi-03-303] Kinlough CL McMahan RJ Poland PA 2006 Recycling of MUC1 is dependent on its palmitoylation. J. Biol. Chem 281 17 12112 22 16507569 10.1074/jbc.M512996200

[b16-bmi-03-303] Kirschenbaum A Itzkowitz SH Wang JP 1999 MUC1 Expression in Prostate Carcinoma: Correlation with Grade and Stage. Mol. Urol 3 3 163 8 10851319

[b17-bmi-03-303] Lapointe J Li C Higgins JP 2004 Gene expression profiling identifies clinically relevant subtypes of prostate cancer. Proc. Natl. Acad. Sci. U.S.A 101 3 811 6 14711987 10.1073/pnas.0304146101 PMC321763

[b18-bmi-03-303] Legrier ME de Pinieux G Boye K 2004 Mucinous differentiation features associated with hormonal escape in a human prostate cancer xenograft Br. J. Cancer 90 3 720 7 14760390 10.1038/sj.bjc.6601570 PMC2409592

[b19-bmi-03-303] Levitin F Stern O Weiss M 2005 The MUC1 SEA module is a self-cleaving domain. J. Biol. Chem 280 39 33374 86 15987679 10.1074/jbc.M506047200

[b20-bmi-03-303] Li C Berx G Larsson C 1999 Distinct deleted regions on chromosome segment 16q23–24 associated with metastases in prostate cancer Genes Chromosomes Cancer 24 3 175 82 10451696

[b21-bmi-03-303] Li Y Bharti A Chen D 1998 Interaction of glycogen synthase kinase 3 beta with the DF3/MUC1 carcinoma-associated antigen and beta-catenin. Mol. Cell Biol 18 12 7216 24 9819408 10.1128/mcb.18.12.7216 PMC109303

[b22-bmi-03-303] Ligtenberg MJ Gennissen AM Vos HL 1991 A single nucleotide polymorphism in an exon dictates allele dependent differential splicing of episialin mRNA. Nucleic Acids Res 19 2 297 301 2014168 10.1093/nar/19.2.297 PMC333593

[b23-bmi-03-303] Macao B Johansson DG Hansson GC 2006 Autoproteolysis coupled to protein folding in the SEA domain of the membrane-bound MUC1 mucin. Nat. Struct. Mol. Biol 13 1 71 6 16369486 10.1038/nsmb1035

[b24-bmi-03-303] Mitchell S Abel P Madaan S 2002 Androgen-dependent regulation of human MUC1 mucin expression Neoplasia 4 1 9 18 NCBI www.ncbi.nlm.nih.gov 10.1038/sj.neo.7900194 PMC1503313 11922395

[b25-bmi-03-303] Notredame C Higgins DG Heringa J 2000 T-Coffee: A novel method for fast and accurate multiple sequence alignment. J. Mol. Biol 302 1 205 17 10964570 10.1006/jmbi.2000.4042

[b26-bmi-03-303] Obermair A Schmid BC Packer LM 2002 Expression of MUC1 splice variants in benign and malignant ovarian tumours Int. J. Cancer 100 2 166 71 12115565 10.1002/ijc.10456

[b27-bmi-03-303] O’Connor JC Julian J Lim SD 2005 MUC1 expression in human prostate cancer cell lines and primary tumors. Prostate Cancer Prostatic Dis 8 1 36 44 15477874 10.1038/sj.pcan.4500762

[b28-bmi-03-303] Palmai-Pallag T Khodabukus N Kinarsky L 2005 The role of the SEA (sea urchin sperm protein, enterokinase and agrin) module in cleavage of membrane-tethered mucins. Febs J 272 11 2901 11 15943821 10.1111/j.1742-4658.2005.04711.x

[b29-bmi-03-303] Papadopoulos I Sivridis E Giatromanolaki A 2001 Tumor angiogenesis is associated with MUC1 overexpression and loss of prostate-specific antigen expression in prostate cancer. Clin. Cancer Res 7 6 1533 8 11410487

[b30-bmi-03-303] Ponting CP Schultz J Milpetz F 1999 SMART: identification and annotation of domains from signalling and extracellular protein sequences Nucleic Acids Res 27 1 229 32 PPSearch http://www.ebi.ac.uk/ppsearch/. 9847187 10.1093/nar/27.1.229 PMC148142

[b31-bmi-03-303] Schmid BC Buluwela L Liu Q 2002 Expression of MUC1 splice variants correlates with invasive growth of breast cancer cell lines. Breast Cancer Res. Treat 76 3 211 9 12462382 10.1023/a:1020853900765

[b32-bmi-03-303] Schut IC Waterfall PM Ross M 2003 MUC1 expression, splice variant and short form transcription (MUC1/Z, MUC1/Y.) in prostate cell lines and tissue. BJU Int 91 3 278 83 12581019 10.1046/j.1464-410x.2003.03062.x

[b33-bmi-03-303] Singh AP Chauhan SC Bafna S 2006 Aberrant expression of transmembrane mucins, MUC1 and MUC4, in human prostate carcinomas Prostate 66 4 421 9 16302265 10.1002/pros.20372

[b34-bmi-03-303] Smith JR Freije D Carpten JD 1996 Major susceptibility locus for prostate cancer on chromosome 1 suggested by a genome-wide search Science 274 5291 1371 4 8910276 10.1126/science.274.5291.1371

[b35-bmi-03-303] Thompson EJ Shanmugam K Hattrup CL 2006 Tyrosines in the MUC1 cytoplasmic tail modulate transcription via the extracellular signal-regulated kinase 1/2 and nuclear factor-kappaB pathways. Mol. Cancer Res 4 7 489 97 16849524 10.1158/1541-7786.MCR-06-0038

[b36-bmi-03-303] Truant S Bruyneel E Gouyer V 2003 Requirement of both mucins and proteoglycans in cell-cell dissociation and invasiveness of colon carcinoma HT-29 cells Int. J. Cancer 104 6 683 94 12640674 10.1002/ijc.11011

[b37-bmi-03-303] Uhlen M 2007 Mapping the human proteome using antibodies Mol. Cell Proteomics 6 8 1455 6 17703056

[b38-bmi-03-303] Wei X Xu H Kufe D 2005 Human MUC1 oncoprotein regulates p53-responsive gene transcription in the genotoxic stress response. Cancer Cell 7 2 167 78 15710329 10.1016/j.ccr.2005.01.008

[b39-bmi-03-303] Wei X Xu H Kufe D 2006 MUC1 oncoprotein stabilizes and activates estrogen receptor alpha. Mol. Cell 21 2 295 305 16427018 10.1016/j.molcel.2005.11.030

[b40-bmi-03-303] Wesseling J van der Valk SW Hilkens J 1996 A mechanism for inhibition of E-cadherin-mediated cell-cell adhesion by the membrane-associated mucin episialin/MUC1. Mol. Biol. Cell 7 4 565 77 8730100 10.1091/mbc.7.4.565 PMC275910

[b41-bmi-03-303] Zhang HK Zhang QM Zhao TH 2004 Expression of mucins and E-cadherin in gastric carcinoma and their clinical significance. World J. Gastroenterol 10 20 3044 7 15378790 10.3748/wjg.v10.i20.3044 PMC4576269

